# Retraction: Experimental and theoretical investigation of a mesoporous NiCo_2_O_4_/rGO nanosheet network as a high-performance anode for lithium-ion batteries

**DOI:** 10.1039/d6ra90120d

**Published:** 2026-09-14

**Authors:** Muhammad Inayat Ullah, Tanveer H. Bokhari, Rimsha Aqeel, Saqib Javed, Zahid Abbas, Shakeel Abbas, Atia Khalid, Athar Javed, Shafqat Karim, Rao Tahir Ali Khan, Amjad Nisar, Mashkoor Ahmad

**Affiliations:** a Nanomaterials Research Group, Physics Division PINSTECH Islamabad 44000 Pakistan mashkoorahmad2003@yahoo.com chempk@gmail.com; b Department of Chemistry, Government College University Faisalabad Pakistan; c Theoretical Physics Division, PINSTECH Islamabad 44000 Pakistan; d School of Materials Science and Engineering, Tsinghua University Beijing China; e Department of Physics, University of the Punjab Lahore 05422 Pakistan

## Abstract

Retraction of ‘Experimental and theoretical investigation of a mesoporous NiCo_2_O_4_/rGO nanosheet network as a high-performance anode for lithium-ion batteries’ by Muhammad Inayat Ullah *et al.*, *RSC Adv.*, 2025, **15**, 20321–20329, https://doi.org/10.1039/d5ra01695A.

The Royal Society of Chemistry hereby wholly retracts this *RSC Advances* article due to concerns with the reliability of the data.

Concerns were raised with the Raman spectra in Fig. 2, with duplications both within and between spectra. The authors have said that an incorrectly processed spectrum derived from the NiCo_2_O_4_/rGO dataset was accidently inserted into the panel labeled NiCo_2_O_4_. The authors have not been able to provide raw data that matches the published spectra. The authors subsequently provided a new Raman plot together with the corresponding raw data and requested a correction.

Given the significance of these concerns, the editor has lost confidence that the findings presented in this paper are reliable.

The authors were informed about the retraction of the article. Saqib Javed, Mashkoor Ahmad, Muhammad Inayat Ullah, Rimsha Aqeel, Amjad Nisar and Shakeel Abbas have agreed with the decision; the other authors have not responded.

Signed: Saqib Javed, Mashkoor Ahmad, Muhammad Inayat Ullah, Rimsha Aqeel, Amjad Nisar and Shakeel Abbas

Date: 4th September 2026

Retraction endorsed by Laura Fisher, Executive Editor, RSC Advances

